# Effects of the immediate postpartum insertion of the etonogestrel implant on the development of breastfed infants: Results from a randomized controlled trial

**DOI:** 10.1002/ijgo.70291

**Published:** 2025-06-09

**Authors:** Mariane Nunes de Nadai, Maria Beatriz Martins Linhares, Juliana Cunha de Lima Rodrigues Sisdeli, Lilian Sheila de Melo Pereira do Carmo, Giordana Campos Braga, Leticia Sanchez Ferreira, Silvana Maria Quintana, Carolina Sales Vieira

**Affiliations:** ^1^ Bauru Medical School University of São Paulo Bauru Brazil; ^2^ Department of Neurosciences and Behavioral Sciences, Ribeirão Preto Medical School University of São Paulo Ribeirão Preto Brazil; ^3^ Universidade de Ribeirão Preto Ribeirão Preto Brazil; ^4^ Universidade Federal de Campina Grande Campina Grande Brazil; ^5^ Department of Obstetrics and Gynecology, Ribeirao Preto Medical School University of São Paulo Ribeirão Preto Brazil; ^6^ Universidade Federal de Uberlância Uberlândia Brazil

**Keywords:** etonogestrel implant, infant development, long‐acting reversible contraception, postpartum contraception

## Abstract

**Objectives:**

To evaluate motor, cognitive, language, and social–emotional development in breastfed infants whose mothers received the etonogestrel (ENG) implant either immediately postpartum or at 6 weeks postpartum.

**Methods:**

This was a secondary analysis from a randomized controlled trial involving 100 postpartum women and their infants. Postpartum women were block‐randomized to receive the ENG implant either within 48 h of delivery (early insertion group, *n* = 50) or at 6 weeks postpartum (delayed insertion group, *n* = 50). We focused on infant development assessed at 6–8 months and 12–15 months using the Bayley‐III Scales of Infant and Toddler Development (BSID‐III). The study was conducted at the University Hospital of Ribeirão Preto Medical School, Brazil. Sociodemographic and clinical characteristics were compared using *t* tests and *χ* ^2^ tests. BSID‐III composite scores were analyzed using mixed‐effects linear regression.

**Results:**

A total of 79 infants completed at least one developmental assessment. No significant differences in baseline sociodemographic and clinical characteristics were observed between groups. At 6–8 months, BSID‐III composite scores across all domains were similar between groups. At 12–15 months, the early insertion group had a significantly higher mean motor score compared with the delayed group (108 ± 11 vs. 99 ± 14, *P* = 0.003), but no significant differences were found in the other domains.

**Conclusion:**

Immediate postpartum ENG implant insertion did not negatively impact infant development up to 12–15 months.

**Clinical trial registration:**

This study was registered on https://clinicaltrials.gov/, registration number NCT02469454, Link: https://www.clinicaltrials.gov/study/NCT02469454?term=NCT02469454%20&rank=1#study‐overview; date of registration: June 9, 2015.

## 1 INTRODUCTION

Initiating long‐acting reversible contraceptives, such as contraceptive implants and intrauterine devices, immediately postpartum has been promoted as an effective strategy to reduce unintended pregnancies and shorten interpregnancy intervals.^1^, ^2^, ^3^, ^4^, ^5^, ^6^

The etonogestrel (ENG) contraceptive implant, a highly effective progestogen‐only method, has demonstrated high continuation rates when initiated in the postpartum period.^7^, ^8^ Immediate postpartum insertion of the ENG implant is considered safe, as it does not significantly impact breastfeeding, infant growth, or maternal outcomes compared with delayed insertion (i.e. after 6 weeks postpartum).^6^, ^9^, ^10^, ^11^ As a result, the World Health Organization's (WHO) Medical Eligibility Criteria for Contraceptive Use classifies immediate postpartum ENG implant insertion as category 2, indicating that the advantages generally outweigh any theoretical or proven risks.^12^

Existing evidence suggests that the timing of ENG implantation does not affect infant growth^8^, ^9^, ^10^, ^13^ but, to our knowledge, no study has specifically assessed its impact on infant developmental outcomes. Given that infant development may be influenced by a variety of factors, including epigenetic mechanisms,^14^, ^15^ the potential effect of early postpartum exposure to hormonal contraception remains a concern for both mothers and healthcare providers. Therefore, we aimed to evaluate the motor, cognitive, language, and social–emotional development of breastfed infants whose mothers received either early or delayed postpartum ENG implant insertion.

## 2 MATERIALS AND METHODS

### 2.1 Study design and settings

This study is a secondary analysis from a previously published open‐label, parallel‐group, randomized controlled trial (RCT) that investigated the effects of immediate postpartum insertion of the ENG implant on infant growth (Clinical Trial Registration: NCT02469454).^16^ The original trial was conducted at the Women's Health Reference Center of Ribeirão Preto, a low‐risk maternity ward affiliated with the University Hospital of Ribeirão Preto Medical School, University of São Paulo, Brazil. The study was approved by the Institutional Review Board (Comitê de Ética em Pesquisa do Hospital das Clínicas de Ribeirão Preto, approval number 62798, national code: CAAE‐02897012.1.0000.5440). This secondary analysis was pre‐specified and described in the original RCT protocol.

For this analysis, infant developmental assessments were conducted at the clinical research unit of the University Hospital of Ribeirão Preto Medical School, University of São Paulo. The women were informed in advance that these assessments were part of the trial secondary outcomes, and all provided signed written informed consent before enrollment.

### 2.2 Participants and randomization process

The original RCT included postpartum women aged 18 years or older who had chosen the ENG implant for contraception, had no contraindications to breastfeeding, and delivered healthy, full‐term newborns (gestational age ≥37 weeks) without congenital malformations, with adequate birth weight for gestational age, and normal sucking ability.^16^ For this secondary analysis, developmental assessments were conducted on these breastfed infants at 6–8 and 12–15 months of age.

Postpartum women were block‐randomized using a computer program (https://www.sealedenvelope.com/simple‐randomiser/v1/lists) by one of the researchers (CSV) into one of two groups: (1) the early insertion group, where ENG‐releasing implants (Implanon®, N.V. Organon, Oss, the Netherlands) were inserted within 48 h of delivery, and (2) the delayed insertion group, where the implants were inserted at 6 weeks postpartum.

Further details on the eligibility criteria and the randomization process have been published previously.^16^

### 2.3 Sample size

The sample size was calculated based on the primary outcome of the RCT, which was the average infant weight at 12 months of age.^16^ A clinically relevant difference between groups was defined as ≥10%,^17^ because weight loss of 5%–10% over 6–12 months is generally considered significant. Therefore, a 10% difference in weight at 12 months was set as the minimum detectable difference. Assuming a standard deviation of ±1186 g (1.2 kg) for weight at 12 months,^18^ a sample of 21 mother–newborn pairs per group was required to achieve 80% power at a 5% significance level. To account for potential losses to follow up, 50 mother–infant pairs were included in each group.

### 2.4 Outcomes and assessments

The primary outcome of this secondary analysis was infant development, assessed using the Bayley‐III Scales of Infant and Toddler Development, Third Edition (BSID‐III).^19^ This standardized tool evaluates developmental functioning in children aged 1–42 months across five key domains: cognitive, language, social–emotional, motor, and adaptive behavior. The BSID‐III includes three independent scales (cognitive, language, and motor) and two questionnaires (social–emotional and adaptive behavior).^19^

BSID‐III generates raw scores, scaled scores, composite scores, and centile ranks for each domain. Developmental performance is classified into one of seven categories (extremely low, borderline, low average, average, high average, superior, or very superior) based on the American population.^19^ Composite scores are calculated by comparing the child's performance with that of an age‐matched normative sample. For the cognitive, language, and motor scales, a mean score of 100 (SD ±15) corresponds to average functioning at the 50th centile. Scores below 85 (1 SD below the mean, corresponding to the 16th centile) indicate mild impairment or “at risk” for developmental delay, while scores below 70 (2 SD below the mean, 2nd centile) suggest moderate to severe impairment.^20^

The BSID‐III has demonstrated strong psychometric properties, including validity, reliability, and internal consistency,^21^, ^22^ and has been validated for use among Brazilian infants.^23^, ^24^

We evaluated infants in two visits: at 6–8 and 12–15 months of age. At each visit, an experienced psychologist certified to administer BSID‐III conducted the evaluations, with each session lasting approximately 60 min.

### 2.5 Data analysis

The statistician responsible for the data analysis was blinded to the study's randomization. As the developmental assessments were conducted in a separate location from the growth assessments, not all infants from the original RCT attended this follow up. Therefore, only infants who completed at least one developmental assessment were included in this analysis. To evaluate potential selection bias, we compared clinical and demographic characteristics between participants and non‐participants.

We analyzed the BSID‐III domains as continuous variables (composite scores) and categorical variables. We used *t* tests, *χ* ^2^ tests, or Fisher exact tests, as appropriate, to compare sociodemographic and clinical characteristics between groups. Mixed‐effects linear regression modeling was used to compare composite scores across BSID‐III domains. Some cells contained zero values, so categorical assessments of the BSID‐III were presented descriptively, without merging categories.

All statistical analyses were performed using the Statistical Analysis System (SAS, version 9.4; SAS Institute Inc., Cary, NC, USA). Missing data were excluded from the analysis, and statistical significance was set at a *P* value less than 0.05.

## 3 RESULTS

In the RCT,^16^ 100 infants were enrolled between June 10, 2015 and November 11, 2016, with 50 (50%) infants assigned to the early insertion group and 50 (50%) assigned to the delayed insertion group. Of these, 95 infants (95%) completed the 6‐month visit and 92 (92%) completed the 12‐month visit of the growth assessments.

**FIGURE 1:**
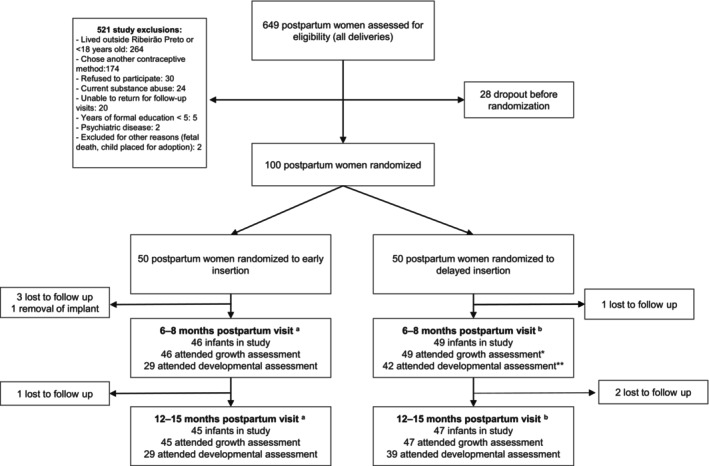
Flowchart of the study. Early represents etonogestrel implant inserted within 48 h of delivery. Delayed represents etonogestrel implant inserted at 6 weeks postpartum. ^a^In the early insertion group, 23 infants attended both developmental assessments (at 6–8 months and 12–15 months), while 12 attended only one (six attended only the 6–8 months visit and six only the 12–15 months visit). ^b^In the delayed insertion group, 37 infants attended both assessments, and seven attended only one (five attended only the 6–8 months visit and two only the 12–15 months visit).

This secondary analysis includes 79 (83.1%) of the 95 infants who attended the 6‐month visit of the growth assessment. Among them, 35 (44.3%) were from the early insertion group and 44 (55.7%) were from the delayed insertion group. In the early insertion group, 23 infants (65.7%) completed both developmental assessments (at 6–8 and 12–15 months), while 12 (34.3%) completed only one (six attended only the 6–8 months visit and six only attended the 12–15 months visit). In the delayed insertion group, 37 infants (84.1%) completed both assessments, and seven (15.9%) completed only one (five attended only the 6–8 months visit and two attended only the 12–15 months visit). A detailed flowchart of attendance is provided in Figure 1.

Table 1 presents the clinical and demographic characteristics of the infants included in this analysis. No statistically significant differences were observed between the groups. Table 2 compares the characteristics of infants who participated in the developmental assessments with those who did not (non‐participants), showing no statistically significant differences.

**TABLE 1:** Clinical and demographic characteristics of the infants who attended the development assessments, whose mothers were randomized to early or delayed postpartum insertion of the etonogestrel implant.^a^

	Early insertion^b^ (*n* = 35)	Delayed insertion^b^ (*n* = 44)	*P* value^c^
Infant sex			0.218
Male	20 (57)	19 (43)	
Female	15 (42)	25 (56)	
Mother's current delivery			0.925
Vaginal	25 (71)	31 (70)	
Cesarean	10 (28)	13 (29)	
Mother's parity			0.653
<3	23 (65.7)	31 (70.5)	
≥3	12 (34.3)	13 (29.6)	
Apgar at 1 min			0.157
<7	2 (5)	7 (16)	
≥7	33 (94)	37 (84)	
Apgar at 5 min			n/a
≥7	35 (100)	44 (100)	
Mother's years of formal education			0.100
2–5	7 (20)	2 (4)	
6–8	12 (34)	18 (41)	
>8	16 (46)	24 (54)	
Attendance at the development assessments			0.111
6–8 and 12–15 months	23 (65.7)	37 (84.1)	
Only 6–8 months	6 (17.1)	5 (11.4)	
Only 12–15 months	6 (17.1)	2 (4.5)	
Gestational age at birth, weeks	39.2 ± 0.3	39.5 ± 1.4	0.263
Birth weight, g	3331 ± 531	3331 ± 403	0.991
Total time of breastfeeding, months	9.4 ± 3.5	9.2 ± 4.2	0.831
Mother's age at childbirth, years	27.2 ± 5.1	25.9 ± 4.9	0.257
Monthly family income, USD	500.3 ± 252	608.7 ± 494.8	0.212

^a^ Data are presented as mean ± standard deviation or number (percentage).

^b^ Early insertion: etonogestrel implants inserted within 48 h of delivery; Delayed insertion: etonogestrel implants inserted at 6 weeks postpartum.

^c^ *P* value obtained by *χ* ^2^ test/Fisher or *t* test.

**TABLE 2:** Comparison of clinical and demographic characteristics between the infants who attended (participants) and those who did not attend (non‐participants) the development assessments.^a^

	Participants^b^ (*n* = 79)	Non‐participants^b^ (*n* = 16)	*P* value^c^
Mother's current delivery			0.739
Vaginal	56 (71)	12 (75)	
Cesarean	23 (29)	4 (25)	
Apgar at 1 min			0.419
<7	9 (11)	3 (19)	
≥7	70 (89)	13 (81)	
Apgar at 5 min			
≥7	77^d^ (100)	15^d^ (100)	
Mother's years of formal education			0.143
2–5	9 (11)	0	
6–8	30 (38)	4 (25)	
>8	40 (51)	12 (75)	
Mother's parity			0.975
<3	54 (68)	11 (69)	
≥3	25 (31)	5 (31)	
Mother's age at childbirth, years	26.5 ± 5	29.1 ± 5.5	0.064
Monthly family income, USD	560.6 ± 408.4	451.9 ± 427.7	0.338

^a^ Data are presented as mean ± standard deviation or number (percentage).

^b^ Participants: infants who attended the development assessments; Non‐participants: infants who did not attend the development assessments.

^c^ *P* value obtained by *χ* ^2^ test/Fisher or *t* test.

^d^ Missing data (two from participants and one from non‐participants).

At 6–8 months of age, the mean composite scores across all BSID‐III domains did not differ significantly between infants in the early and delayed insertion groups (Table 3). However, at 12–15 months of age, the mean motor composite score was 9.1% higher in infants from the early insertion group compared with those from the delayed insertion group (early insertion: 108 ± 11 vs. delayed insertion: 99 ± 14, *P* = 0.003). The other BSID‐III domains showed no significant differences between the groups at this age (Table 3).

**TABLE 3:** Effect of early postpartum insertion of etonogestrel implants on the development assessments using Bayley‐III Scales in infants at 6–8 and 12–15 months of age.^a^

Development domains	6–8 months of age			12–15 months of age		
	Early insertion^b^ (*n* = 29)	Delayed insertion^b^ (*n* = 42)	*P* value^c^	Early insertion^b^ (*n* = 29)	Delayed insertion^b^ (*n* = 39)	*P* value^c^
Cognitive	108 ± 9 90–130	111 ± 9 90–130	0.328	113 ± 10 85–130	108 ± 12 80–130	0.063
Language	120 ± 8 100–129	119 ± 9 103–129	0.588	109 ± 7 94–121	105 ± 7 89–121	0.053
Motor	112 ± 10 88–130	110 ± 12 88–133	0.533	108 ± 11 82–121	99 ± 14 70–130	0.003
Social–emotional	115 ± 9 90–130	113 ± 11 80–130	0.269	117 ± 12 95–140	112 ± 12 85–140	0.067

^a^ Data are presented as mean ± standard deviation (SD) and range. A mean score of 100 (SD ±15) indicates mid‐average functioning, a mean score below 85 (1 SD below the mean) indicates mild impairment of being “at risk” of developmental delay, and a mean score below 70 (2 SD below the mean) indicates moderate to severe impairment.^20^

^b^ Early insertion: etonogestrel implants inserted within 48 h of delivery; Delayed insertion: etonogestrel implants inserted at 6 weeks postpartum.

^c^ *P* value obtained by mixed‐effects linear regression models.

A descriptive assessment of the BSID‐III domains (cognitive, language, motor, and social–emotional) revealed that all infants scored within the low average to very superior range at both 6–8 and 12–15 months of age. No infants were classified as extremely low or borderline in any domain at any assessment point (Table 4).

**TABLE 4:** Description of development assessments using Bayley‐III Scales in infants at 6–8 and 12–15 months of age whose mothers were randomized to early or delayed postpartum insertion of the etonogestrel implant.^a^

Development domains	6–8 months of age		12–15 months of age	
	Early insertion^b^ (*n* = 29)	Delayed insertion^b^ (*n* = 42)	Early insertion^b^ (*n* = 29)	Delayed insertion^b^ (*n* = 39)
Motor				
Low average	1 (3)	1 (2)	2 (6)	11 (21)
Average	6 (21)	17 (40)	11 (38)	19 (49)
High average	15 (52)	14 (33)	13 (45)	4 (10)
Superior	6 (21)	7 (17)	3 (10)	4 (10)
Very superior	1 (3)	3 (7)	0 (0)	1 (3)
Cognitive				
Low average	0 (0)	0 (0)	2 (6)	2 (5)
Average	10 (25)	15 (36)	5 (17)	12 (31)
High average	17 (59)	20 (48)	13 (45)	19 (49)
Superior	1 (3)	6 (14)	8 (28)	4 (10)
Very superior	1 (3)	1 (2)	1 (3)	2 (5)
Language				
Low average	0 (0)	0 (0)	0 (0)	1 (3)
Average	5 (17)	11 (26)	15 (52)	25 (64)
High average	6 (21)	9 (21)	13 (45)	12 (31)
Superior	18 (63)	22 (52)	1 (3)	1 (3)
Very superior	0 (0)	0 (0)	0 (0)	0 (0)
Social–emotional				
Low average	0 (0)	2 (5)	0 (0)	1 (3)
Average	4 (14)	6 (14)	6 (21)	12 (31)
High average	12 (41)	23 (55)	9 (31)	16 (41)
Superior	11 (38)	7 (17)	9 (31)	5 (13)
Very superior	2 (6)	4 (10)	5 (17)	5 (13)

^a^ Data are presented as number (percentage).

^b^ Early insertion: etonogestrel implants inserted within 48 h of delivery; Delayed insertion: etonogestrel implants inserted at 6 weeks postpartum.

## 4 DISCUSSION

Our study found no significant differences in developmental outcomes up to 12–15 months of age among breastfed infants whose mothers received the ENG implant either immediately postpartum or at 6 weeks postpartum. Notably, the only statistically significant finding was a 9.1% higher mean composite score for motor development in the early insertion group compared with the delayed insertion group at 12–15 months. However, this difference may not be clinically meaningful based on the standard interpretation of BSID‐III scores.^20^

Cognitive development in infants involves the progressive acquisition of skills related to perception, attention, memory, language, problem‐solving, and reasoning. Although genetics, environment, and early experiences are critical determinants, hormonal influences also play a significant role. Sex hormones such as testosterone and estrogen exert organizational effects on brain development and cognitive functions, particularly during prenatal and early postnatal periods. These hormones contribute to sex‐related differences in cognitive abilities by influencing brain differentiation and organization.^24^, ^25^

Although some studies have suggested a relationship between hormonal exposure and cognitive function,^25^ previous research found no differences in the development of infants breastfed by women who received either an ENG implant or a copper intrauterine device inserted 28–56 days postpartum.^13^ Another study comparing the 52‐mg levonorgestrel intrauterine device to the copper intrauterine device inserted 6–8 weeks postpartum in lactating women also reported no differences in infant developmental outcomes.^13^, ^26^ Consistent with these studies,^13^, ^26^ our study showed no negative effects on infant development in relation to the timing of postpartum ENG implant insertion. These results contribute to the growing body of evidence supporting the safety of immediate postpartum ENG implant use, which has also been shown not to impair breastfeeding or infant growth outcomes.^8^, ^10^, ^11^, ^12^, ^13^ Our findings further reinforce the suitability of this contraceptive method during the early postpartum period, addressing a common concern among both patients and healthcare providers.

The strengths of our study include its design as a randomized clinical trial and the use of a well‐established developmental assessment tool, the BSID‐III. Unlike other tools that may focus on only one domain (e.g. cognitive or motor skills), the BSID‐III provides a comprehensive evaluation across five key domains: cognitive, language, motor, social–emotional, and adaptive behavior. Its detailed and holistic approach gives it an advantage over other developmental assessment tools.^20^, ^24^ Additionally, the BSID‐III was administered by a trained professional in our study, enhancing the reliability of our findings. To our knowledge, no previous study on postpartum contraception has assessed infant development using the BSID‐III. Although the Bayley scale has since been revised to its fourth edition (BSID‐IV),^27^ the third edition was the current version at the time of our study.

Despite its strengths, our study has limitations. First, this was a secondary analysis of an RCT originally powered to evaluate infant growth.^16^ As a result, the study may not have had sufficient statistical power to detect subtle differences in developmental measures, and non‐significant findings should be interpreted with caution. Additionally, about 18% of infants who participated in the growth assessments did not attend the developmental assessments. Although this level of attrition is below the 20% threshold often considered problematic in RCTs,^28^, ^29^ it may still introduce bias. The discrepancy in participation rates likely reflects logistical challenges, as the developmental assessments were conducted at a different location and required a longer and more detailed evaluation. To mitigate potential bias, we compared clinical and socioeconomic characteristics between infants who did and did not participate in the developmental assessments and found no significant differences between groups. Another limitation is that our developmental assessment was limited to infants up to 15 months of age, preventing us from making conclusions about the long‐term effects of early progestogen exposure on infant development. Future studies with longer follow up are needed to evaluate these outcomes more comprehensively.

In conclusion, our study found no significant differences in developmental outcomes up to 12–15 months of age between breastfed infants whose mothers had early versus delayed postpartum ENG implant insertion. Expanding the safety data on immediate postpartum initiation of the ENG implant could enhance its acceptability among both patients and healthcare providers.

## AUTHOR CONTRIBUTIONS

CSV, LS, SMQ, GCB, MNN, JCLRS, and MBML substantially contributed to the conception and design of the project. JCLRS and MBML collected infant development data using Bayley's scale. All authors contributed to the analysis and interpretation of the data, drafting of the manuscript, critical revision of the intellectual content, and giving final approval of the version to be published.

## CONFLICT OF INTEREST STATEMENT

CSV serves on Medical Advisory Boards and provides lectures for Bayer, Exeltis, and Organon. MNN provides lectures for Bayer, Exeltis, and Organon. GCB provides occasional lectures for Organon. The remaining authors have no conflicts of interest.

## Supporting information


Data S1.

## Data Availability

The data that support the findings of this study are available from the corresponding author upon reasonable request.
